# Police officers’ perspective on doping and prevention among recreational athletes: a cross-sectional study

**DOI:** 10.3389/fspor.2023.1251531

**Published:** 2023-10-23

**Authors:** Pia Kvillemo, Johanna Gripenberg, Anna K. Strandberg, Tobias H. Elgán

**Affiliations:** Department of Clinical Neuroscience, STAD, Centre for Psychiatry Research, Karolinska Institutet, and Stockholm Health Care Services, Region Stockholm, Stockholm, Sweden

**Keywords:** anabolic androgenic steroids, substance use, public health, police intervention, recreational athletes, multi-component program

## Abstract

**Introduction:**

The use of anabolic androgenic steroids among recreational athletes has received growing attention in recent decades. Several countries have implemented bans on doping; however, recreational athletes and other subpopulations continue to use doping substances. Recognizing that the police play a crucial role in preventing the use and dealing of doping substances in Sweden, efforts have been made to intensify police interventions and enhance collaboration with other key actors. This study examined police officers’ perceptions of doping as defined in Swedish law, related problems, and suggestions for effective prevention of doping in the society.

**Methods:**

A cross-sectional survey study was conducted using a web survey of police officers (*N* = 597). Data were analyzed using descriptive statistics and free-form text responses were analyzed using content analysis.

**Results:**

Participant responses to the survey (73.7% response rate) indicated that approximately 62.6% thought that doping is a societal problem, and approximately 26% perceived that the availability of doping substances has increased over the past three years. A total of 95.6% of respondents believed that doping occurred in connection with other crimes such as intimate partner violence (88.2%) and drug-related crimes (88.0%). Further, 96.3% of respondents perceived that it was their duty to prevent doping, but 63.8% indicated that doping-related work was not prioritized within their local police district.

**Discussion:**

Police officers perceived doping as a societal problem and expressed motivation to counteract it, highlighting increased knowledge, legislative changes, intensified doping prevention in gyms, and commitment from other societal actors to increase the effectiveness of doping prevention. Suggestions for increasing the efficiency of doping prevention included education and increased knowledge at all levels in the police organization, intensified prevention efforts at gyms, legislative changes to permit simplified doping test procedures, and breach of secrecy for postal items. There was also a suggestion for further engagement from other actors, such as healthcare workers, school officials, and non-governmental organizations.

## 1. Introduction

In recent decades, the use of anabolic androgenic steroids (AAS) has attracted attention as a growing phenomenon among recreational athletes, such as body builders (1–8). AAS is one of several performance-enhancing drugs and is a doping substance if not used with a physician's prescription for medical purposes. In international competitive sports, the use of AAS and other substances aimed to increase athletes’ performance is regulated by the World Anti-Doping Agency in accordance with a list of substances that are not allowed in the body of participants in elite sports contests (9). The regulation of doping substances in recreational contexts, however, differs between countries—specifically, the criminalization of their manufacturing, handling and use, and which doping substances are deemed illegal (10).

Users of doping substances are at risk of negative physical and mental health effects. Specifically, the use of AAS is associated with aggression, depression, infertility, liver and muscle damage, cardiac injuries, arrhythmias, and sudden cardiac death (11–20). In addition to individual health problems, AAS is associated with users’ violence and crimes against other (21, 22). The prevalence of doping outside the elite sports context is difficult to measure; however, estimates from Western countries suggest that approximately 1% of national populations use doping, comprised largely of men (23, 24). Self-reported data from recreational athletes and other subpopulations, however, show higher rates (3, 25). In a Nordic meta-analysis on the lifetime prevalence of non-medical use of AAS, the authors found a lifetime prevalence of 59.2% among drug users and 26.2% among prisoners and arrestees (3). Furthermore, prevalence estimates among recreational athletes in eight European countries indicated that 3.1% of men and no women reported doping in 2019 (26). Finally, self-reported data on AAS use among gym-goers in Stockholm County (Sweden) showed a lifetime prevalence of 3.9% among men, while 1.4% of them had used AAS during the past 12 months (27).

To counteract health and social problems associated with doping substances, Sweden adopted its first doping law in 1991 (28). The act on the prohibition of certain doping substances covers synthetic anabolic steroids and testosterone, and its derivatives; growth hormone and chemical substances that increase the production and release of either testosterone and its derivatives or growth hormone (24, 28). In Sweden, in contrast to most other countries, not only are the manufacturing, sale, supply, and possession of doping substances criminalized, but the use of them outside the healthcare sector is also criminalized, which implies that detection in the human body can lead to punishment (6, 10, 28). The implementation of the Swedish doping law amplified anti-doping work in the recreational sports context from the 1990s onwards (6). Despite these efforts, the ban has proven insufficient to prevent the use of doping substances in recreational sports (27). To increase the possibility of preventing doping among recreational gym-goers in Sweden, a multi-component program—100% Pure Hard Training (PHT)—was developed in 2007 (29). The program has since then been disseminated nationwide and is currently used in around 600 gyms across Sweden. Within the program, the police officers are crucial actors who can enforce the law through supervision at gyms.

Owing to concerns about recreational athletes’ use of doping, the European Union (EU) Commission published a review of the evidence base for policies to combat doping in recreational sports in 2014 (10). Along with legislative measures and controls, this review concluded that doping prevention in recreational sports relies primarily on education and information. The authors explained that published studies examining the effects of anti-doping education programs are rare. Some experts who contributed to the review claimed that educational media campaigns could have the intended effects; however, since most legal, administrative, and political arrangements regarding doping prevention in recreational sports were fairly new in 2014, the report by the EU Commission could not present a consensus on ideal practices (10). Later, a single study obtained support for combined educational programs and practical strength training, targeting adolescents and students (30). Another exploratory study suggested that prevention (educational) efforts should involve key stakeholder groups, such as coaches (31). In 2021, Bates and Vinther conducted a literature review on doping prevention interventions and concluded that the evidence base remained underdeveloped and the effects unclear (4).

Given the important role that the police plays in preventing doping in recreational sports in a Swedish context, particularly at gyms, this study examines police officers’ perceptions of doping, as it is defined in Swedish law, related problems, and suggestions for effective prevention of doping in the society. This study can contribute to development of the knowledge base concerning recreational doping and how to prevent it, as national regulations are continuously changing based on research and practice.

## 2. Methods

### 2.1. Design

This study used a cross-sectional approach, based on a web survey of employees within the police authority in Sweden.

### 2.2. Participants and procedure

Participants were recruited in three different ways. Initially, three police officers with a coordinating function regarding substance use-related issues (including doping) and crime prevention invited police officers who had previously attended a two-hour lecture on doping and colleagues who had not attended any known training on doping. The second sample consisted of police officers who signed up for a half day of digital training in police-related doping prevention work, which was provided within the 100% PHT program. Finally, the possibility of participating in this study was communicated through the police authority's own internal channels: the Swedish Narcotics Police Association's magazine and the 100% PHT website. Therefore, the sample was a convenience sample. Potential participants were invited via e-mail and informed about the study purpose and other relevant aspects connected to the Declaration of Helsinki. The e-mail also contained a link to the web survey (Questback), which the participants could complete after providing informed consent to participate. The survey was based on previous prevention and implementation research (32–34), and it was developed by STADs’ researchers in consultation with selected police officers and doping prevention coordinators. The questions covered participants’ backgrounds, doping as a societal problem, availability of doping substances, and police's doping prevention activities, along with related barriers and possibilities. The questions had fixed answer options; however, comments and elaborate answers were left in the free-text area following several questions. In addition, free-text questions were included. The survey was pilot tested by police officers and took about 15–20 min to complete.

A total of 810 police officers were invited to answer the survey, and data were collected between October 2020 and December 2021. To increase the response rate, several reminders were sent to those who had not yet completed the survey. Data were analyzed using SPSS 27.0 (IBM, Armonk, NY, USA) and presented descriptively. The free-text answers were analyzed using qualitative content analysis. The study was performed in accordance with the Declaration of Helsinki, and the protocol was sent to the Swedish Ethical Review Authority (no. 2019-05156).

### 2.3. Analysis

Quantitative data were analyzed using SPSS 27.0 (IBM, Armonk, NY, USA) and presented descriptively. Some questions had minor internal non-responses as reported in the respective tables. Mann–Whitney *U*-tests were used for comparative analyses based on median values and Chi-square tests (*χ*^2^) were used to compare proportions. For all analyses, a *p*-value of < .05 was considered a statistically significant difference. The qualitative material was analyzed using directed qualitative content analysis (35), focusing on the literal meaning of words (manifest analysis.) One author (TE) repeatedly read the free-text answers to group the content into meaningful categories. Four categories were generated and further discussed with a second author (PK), who agreed on the number of categories, which were named “general action,” “action on gyms,” “the police's work to curb doping,” and “barriers to police's doping prevention activities.”

## 3. Results

### 3.1. Participants

A total of 597 police officers completed the survey (73.7% response rate). The median age was 35 years (interquartile range 30-41, range 22-64), and a majority of participants were men. Almost half of the respondents had worked as a police officer for eight years or more, and most had worked as either intervention or community police officers (Table 1). Respondents represented all seven police regions in Sweden and almost half reported that they had some form of previous doping-related education. Further, almost half had heard of 100% PHT.

**Table 1:** Background information of respondents (*N* = 597).

Sex, % (*n*)^b^	
Female	33.3 (198)
Male	66.7 (397)
Number of years as police officer, median, interquartile range, range^a^	6, 3–12, 0–45
Number of years as police officer in categories, % (*n*)	
0–3	29.3 (174)
4–7	25.6 (152)
8–12	21.4 (127)
12	23.6 (140)
Role, % (*n*)	
Intervention police officer	44.9 (268)
Community police officer	32.0 (191)
Municipality police officer	5.5 (33)
Investigating officer	4.7 (28)
Undercover police officer	4.0 (24)
Investigation leader	3.2 (19)
Other	5.7 (34)
Police region, % (*n*)^c^	
Stockholm	23.2 (136)
Öst	21.7 (127)
Nord	20.6 (121)
Syd	12.5 (73)
Bergslagen	9.4 (55)
Väst	9.2 (54)
Mitt	3.4 (20)
Previously received training on doping? % (*n*)^d^	
Yes, during undergraduate studies	26.8 (160)
Yes, through continuing education	11.9 (71)
Yes, in a different way	10.6 (63)
No	50.7 (302)
Have you heard about the method 100% pure hard training? % (*n*)^e^	
Yes	48.9 (215)
No	51.1 (225)

Missing data: ^a^*n* = 4, ^b^*n* = 2, ^c^*n* = 11, ^d^*n* = 1, ^e^*n* = 157.

Since 38% (*n* = 227) had partaken in a previous two-hour educational course and 62% (*n* = 370) had not, comparative analyses were made between the two sub-samples. There was no significant difference in median age (*z* = −1.899, *p* = .058) nor sex (*χ*^2^(1) = 0.569, *p* = .474) between the two sub-samples. However, there was a difference in the number of median years the two samples had worked as police officers as the median value was 10.0 years (interquartile range = 3.0–14.0) for those who had partaken in the course and 6.0 years (interquartile range = 3.0–12.0) for those who had not (*z* = −2.400, *p* = .016). Regarding the two sub-samples’ view on doping as a societal problem, there was no difference (*χ*^2^(4) = 7.784, *p* = .100). Since the two samples were similar with respect to all but one of these variables, they are treated as one sample henceforth.

When comparing our sample distribution from the distribution of the police force as a whole (data derived per 2023-07-31 from human resources department centrally at the police, personal communication), there was no difference in the proportion of women (33% vs. 34%, *z* = 0.367, *p* = .711). Regarding the region where respondents work, the sample distribution from Bergslagen (9.4%) and Stockholm (23.2%) was the same as the police force as a whole (7.3% and 23.4%, respectively; *z* = −1.883, *p* = .060 and *z *= 0.081, *p* = .936), but there was an overrepresentation of respondents from Nord (20.6% vs. 7.9%, *z *= −11.112, *p* < .001) and Öst (21.7% vs. 8.7%, *z* = −10.770, *p* < .001). Regions with an underrepresentation of respondents were region Mitt (3.4% vs. 7.6%, *z* = 3.829, *p* < .001), Syd (12.5% vs. 18.6%, *z* = 3.779, *p* < .001), and Väst (9.2% vs. 18.1%, *z* = 5.523, *p* < .001).

### 3.2. Doping as a societal problem

More than 60 percent of the respondents believed that doping was a large or very large societal problem (Table 2). Almost all respondents thought that it was their duty to prevent doping, which some respondents underlined by commenting that doping is *de facto* illegal. Moreover, most reported that they met people who use, or whom they suspect use, doping substances once a month or more often, and almost half reported similar figures for the handling of doping substances (Table 2). However, several respondents commented that the unrecorded number was probably large because of the lack of targeted efforts against doping and a lack of knowledge.

**Table 2:** Perception of doping and the police's work to curb doping (*N* = 597).

Do you think doping is a problem in society? % (*n*)	
Yes, a very large problem	12.2 (73)
Yes, a large problem	50.4 (301)
Neither large nor small problem	23.6 (141)
Fairly small problem	6.5 (39)
Don't know	7.2 (43)
Do you think it is part of your duties to prevent doping? % (*n*)^a^	
Yes	96.1 (574)
No	1.8 (11)
Not relevant to my role	1.8 (11)
Do you in your work meet people who use, or who you suspect have used doping substances? % (*n*)	
Several times per week	2.8 (17)
Sometime per week	12.7 (76)
Sometime per month	38.9 (232)
Sometime per year	38.5 (230)
Never	1.3 (8)
Not relevant to my role	3.2 (19)
Don't know	2.5 (15)
Do you in your work meet people who deal with, or who you suspect deal with doping substances? % (*n*)	
Several times per week	2.7 (16)
Sometime per week	8.7 (52)
Sometime per month	34.7 (207)
Sometime per year	42.9 (256)
Never	3.0 (18)
Not relevant to my role	3.2 (19)
Don't know	4.9 (29)
In connection to which other types of crime do you think doping occurs? % (*n*)^a^	
Violent crimes	95.6 (568)
Intimate partner violence	88.2 (524)
Drug-related crime	88.0 (523)
Gang-related crime	85.7 (509)
Robbery, assault	57.1 (339)
Sexual offenses	54.7 (325)
Juvenile delinquency	42.8 (254)
Vandalism	32.3 (192)
Traffic offenses	29.8 (177)
Burglary	15.2 (90)

Missing data: ^a^*n* = 3.

Several respondents commented that doping occurs in connection with other crimes (Table 2) and has become a very common feature in the lifestyle of gang criminals. Almost all participants (95.6%) thought that doping occurred in connection with violent crimes. The corresponding figures for intimate partner violence and drug-related crimes were each more than 80 percent (Table 2). The perception that doping is overlooked several times, especially in intimate partner violence, was also reported by several participants.

### 3.3. Availability of doping substances

Approximately one-fourth of the participants perceived that the availability of doping substances had increased to a certain extent during the last three years (Table 3). The respondents commented that this was mainly owing to the increased availability on the Internet, in which substances are sold on encrypted sites.

**Table 3:** Availability of doping substances and the prevalence of doping-related problems (*N* = 597).

Do you think that the availability of doping substances has increased in the last three years? % (*n*)^a^	
Large increase	6.4 (38)
Some increase	19.7 (117)
No difference	16.3 (97)
Some decrease	0.3 (2)
Large decrease	0.2 (1)
Don't know	57.1 (339)
Do you experience in your work that the occurrence of doping-related problems has changed in the last three years? % (*n*)^b^	
Large increase	4.7 (28)
Some increase	20.8 (124)
No difference	25.8 (154)
Some decrease	2.9 (17)
Large decrease	0.2 (1)
Don't know	45.6 (272)
Where do you think it is most common for users to get hold of doping substances for the first time? % (*n*)^a^	
The internet	53.9 (320)
Gym and training facilities	22.4 (133)
Friends	21.5 (128)
Other	2.2 (13)

Missing data: ^a^*n* = 3, ^b^*n* = 1.

Further, respondents commented that the detection of postal items with doping has increased, which they indicated might be owing to postal companies becoming more attentive. When asked if they experienced a change in the incidence of doping-related problems during the last three years, approximately one-fourth of respondents answered that they felt there had been an increase to some or a large extent (Table 3). Comments from respondents revealed the perception that doping has become more common among younger people and that it is commonly used by criminals, not least by gang criminals. However, some participants emphasized in comments that increased knowledge about doping also contributes to the perception that doping has increased. Several also indicated that they perceived that a correct assessment of potential users is difficult to carry out because many who use doping do not necessarily have large muscles; thus, the police may not have reason to suspect them.

Just over half the respondents reported that they thought the Internet was the most common way for doping users to obtain doping substances for the first time (Table 3). Approximately one in five respondents reported that they believed first-time users are introduced to doping through friends and at gyms.

### 3.4. Prerequisites for police work against doping

Most respondents thought that the issue of doping was not a priority in their local police district (Table 4). Further, just over half of the respondents stated that they were not given the necessary prerequisites to work toward decreasing doping, and just over one-third stated that they were partially given these prerequisites. Reasons for this, such as resource and knowledge gaps in the organization, were also provided in comments. Almost three out of four respondents stated that they lacked further training in their anti-doping work, about 60 stated that there was a lack of human resources, and approximately 30 stated that supervision was missing (Table 4). They also commented on the need to clarify the link between doping and other crimes and opined that many police officers lack knowledge about how to present evidence for offences that can lead to prosecution. Accordingly, the importance of raising the level of knowledge among investigation leaders so they can make confident decisions that succeed in court was proposed by some participants. Several participants also commented that police officers, including themselves, had to attend doping training to highlight the doping issue. Some said they have trained officers in their local police district who act as liaison officers for gyms. Just over half the respondents requested a simplified procedure in doping tests, stating that blood tests or other rapid tests could be used. This suggestion was put forward due to the consideration that the collection of urine samples is sometimes difficult, especially if necessary permission from the investigation leaders is not given or if the suspect refuses to provide the sample (Table 4).

**Table 4:** Prerequisites for police work against doping (*N* = 597).

Do you think that doping is prioritized in your local police district? % (*n*)	
Yes	5.0 (30)
Partly	23.3 (139)
No	63.8 (381)
Not relevant to my role	0.3 (2)
Don't know	7.5 (45)
Do you think that your local police district work strategically with doping? % (*n*)	
Yes	21.1 (126)
No	61.1 (365)
Don't know	17.8 (106)
Do you think that you are given sufficient resources to work against doping? % (*n*)	
Yes	7.5 (45)
Partly	35.5 (212)
No	53.3 (318)
Not relevant to my role	3.7 (22)
Are you missing something in your work against doping? % (*n*)^a^	
Training/knowledge	73.6 (420)
Personnel resources	57.1 (326)
Simplified procedures for doping tests	56.0 (320)
Collaboration	32.4 (185)
Supervision	29.9 (171)
Doping issue should be mandatory in the undergraduate program	38.9 (165)
Support from my closest chief	11.0 (63)
Economic resources	9.8 (56)
To what extent do you in your local district police work with inspections at gyms? % (*n*)^b^	
Sometime per week	1.0 (6)
Sometime per month	10.6 (63)
Sometime per year	34.1 (202)
Never	26.8 (159)
Don't know	27.5 (163)

Missing data: ^a^*n* = 26, ^b^*n* = 4.

Many respondents reported that their local police districts have targeted efforts directed at gyms once or twice per year. Some respondents commented that these interventions have been successful but also perceived as insufficient (Table 4). Just over one-third of respondents reported that such targeted efforts happen once a year, and approximately one-fourth reported that it never happens. About one in ten respondents, however, reported that it occurred only once a month (Table 4).

### 3.5. Cooperation with other actors

Almost one-third of respondents stated cooperation was lacking in their doping work (Table 4), while one-third reported that they collaborated with 15 other actors regarding doping issues (about 40% were unaware of collaboration; Table 5). By far, the most common actors that the police collaborate with are gyms and training facilities, but they also collaborate with municipal coordinators and the County Administrative Board to some extent. Further, 40 mentioned that they believed that doping is included in the citizens’ pledge between the police authority and the municipality, while just over one-third did not think so (Table 5). In addition to the police authority, almost all respondents reported that they believed that gyms and training facilities should engage in anti-doping work to obtain success (Table 5). A large proportion of respondents further suggested that several other actors, such as the healthcare workers, school officials, post office workers, youth clinic workers, social service employees, customs officers, and municipal coordinators, should engage in doping prevention. Other actors that were mentioned in the comments were sports association and other non-governmental organizations.

**Table 5:** Collaboration in doping prevention with other actors (*N* = 597).

Do you in your local district police collaborate with other actors concerning doping issues? % (*n*)	
Yes	35.5 (212)
No	20.9 (125)
Don't know	43.6 (260)
What other actors do you collaborate with? % (*n*)^a^	
Gyms and other training facilities	94.2 (195)
Municipal coordinators	31.4 (65)
County administrative board	22.2 (46)
Schools	17.4 (36)
Social services	16.9 (35)
Postal agents	10.1 (21)
Healthcare	9.2 (19)
Customs	8.2 (17)
The Swedish sports confederation	6.3 (13)
Do you think that citizens’ pledge between the police authority and the municipality is an important prerequisite to conduct work against doping? % (*n*)^b^	
Yes	40.3 (240)
No	34.6 (206)
Don't know	25.2 (150)
What other actors besides the police authority do you think should engage in work against doping for it to be efficient? % (*n*)^c^	
Gyms and other training facilities	95.4 (557)
Healthcare	73.6 (430)
Schools	72.4 (423)
Postal agents	58.6 (342)
Youth health clinics	58.4 (341)
Social services	58.0 (339)
Customs	57.9 (338)
Municipal coordinators	50.0 (292)
County administrative board	30.3 (177)
The Swedish sports confederation	25.5 (149)
Non-governmental organizations	20.2 (118)

Missing data: ^a^*n* = 5 (could only be answered by 212 respondents), ^b^*n* = 1, ^c^*n* = 13.

Regarding postal agents, some participants mentioned that the current legislation is a barrier to doping prevention, as the penalty value is not high enough for secrecy in the Postal Act to be breached. The Postal Act contains provisions concerning postal operations and universal postal service.

### 3.6. Reflections on doping prevention work

Finally, the respondents were asked to reflect on what they thought could be done to prevent doping in society. Over half the respondents contributed with reflections, which were qualitatively analyzed and interpreted, and subsequently divided into the following themes: general actions, actions at gyms, police work to curb doping, and barriers to police doping prevention activities.

#### 3.6.1. General actions

Several respondents referred to the fact that young men today obtain their masculine ideals from the media, not least from social media, in which they are given an image of a perfect male body with six-pack abs. Thus, they meant that many men strive for this ideal body and search for quicker ways to attain it.

*Society as a whole has a twisted image of the male ideal where the man is portrayed as a 25-year-old with a six-pack and big arms/shoulders in every other commercial for all kinds of products. Visible muscles indicate success*.

Some respondents suggested that information efforts in schools and training facilities could be an appropriate action to counteract the influence of social media by informing students and the public about the consequences of doping, physically and legally.

*Inform schools and training facilities about the risks. Today, there is an incredible obsession with how you should look and that you should have quick results. Remind young people that you cannot aspire to look like celebrities and others on social media. One way is to involve social media profiles/influencers in the work against doping. We also need more inspections on training facilities and more effective prosecution*.

Similarly, it was suggested that sports associations inform young members about doping.

*Talk about it in schools and sports associations and highlight the consequences of doping*.

According to respondents, if suspicion exists about doping use, teachers or leaders should talk to the person individually.

*Schools should also talk about the effects of doping and, if suspected, talk privately with the concerned person*.

Some respondents also mentioned that the level of knowledge among staff in healthcare settings, including psychiatry, needs to be raised to become better at detecting and dealing with people subject to doping.

*More people in healthcare and psychiatry who are trained and perceptive to the side effects of doping, such as depression, potency problems, aggression, heart problems [should be engaged]*.

#### 3.6.2. Actions at gyms

Several respondents highlighted the importance of more gyms becoming involved in and actively working against doping.

*Gyms [should] clearly distance themselves from doping*.

It was further mentioned that gyms, to manifest their policy, need to communicate with their members that they take an active stance against doping; there is visible information in gyms about the consequences of doping; the gyms work with policies and clear procedures for how they manage various doping-related situations; they train their staff in the doping issue; and they cooperate with the police when doping is suspected (e.g., by tipping off the police). Suggestions were also made that gyms could have mandatory talks with young people younger than 18-years-old who buy a gym membership card, informing them about doping, and what regulations the gym applies regarding the use of doping substances.

*Gyms should possibly “force” young people under the age of 18 who buy membership from them to participate in an information meeting about anabolic androgenic steroids*.

Further, it was suggested that the membership agreement between the customer and the gym should state that as a member, one approves testing in the event of suspected doping or routine check, and that if one fails to take samples or test positive, they will be suspended from the gym until clean results are provided.

*They [the gyms] should be able to demand a urine sample if they have their own suspicion. It should be stated in the agreement between the customer and the gym that it is 100% Pure Hard Training and in case of suspicion, you have signed in the agreement/membership that you accept this*.

Several participants also mentioned the doping prevention method of 100% PHT, and that it is a well-established method that gyms should work with. Further, several participants believed there was a need for good collaboration with gyms to facilitate more frequent and targeted supervisory visits. It was also mentioned that the local police district should appoint liaison officers who are trained in the doping issue and work more closely with the gyms, for example through agreements, to promote cooperation between gyms and the police and enable undercover visits.

#### 3.6.3. Police’s work to curb doping

In addition to what was mentioned about how the police can work by targeting more gyms and other such facilities, other proposals for more effective doping prevention were mentioned by the respondents. Primarily, these are about increasing the knowledge level among police officers because doping is often connected to other crimes. Some mentioned that doping should be a compulsory element in basic education at police academies.

*Make the debriefing clearer, introduce it as a mandatory part of basic [police] education, and increase knowledge internally [in the police organization]*.

The importance of increasing knowledge among investigation leaders was highlighted as critical because their decisions is needed to collect and present evidence of doping offenses. Respondents also mentioned the need for the police to work more systematically with targeted efforts in the same way as it did for the campain “traffic weeks” in an attempt to counteract vehicle accidents. Finally, participants mentioned that obtaining doping substances often takes place on the Internet, and that it is desirable to have targeted efforts with systematic Internet spying to access suppliers.

*The police authority must invest resources in targeting [doping] suppliers* via *Internet surveillance*.

#### 3.6.4. Barriers to the police’s doping prevention activities

A recurring barrier to doping prevention activities that was reported by the respondents, and outlined above, is the low level of knowledge about doping within the police force, prosecutors, and the “judicial ombudsmen” (JO)—an authority that examines that other authorities work in accordance with the laws and regulations that govern their work. One respondent described a case that began with suspected drunk driving, which was then extended to doping offenses after taking the offender into custody because of clear signs of intoxication. This was judged to be a reasonable basis for the decision to search the offender's house. During the search, doping substances were found. However, the case was, according to the respondent, reported to the JO, who assessed that the circumstances leading to the decision to search the house were insufficient for a reasonable suspicion that the suspect had doping substances at home, which meant that there was no basis for the decision to search the house.

*Even though we had found doping [in the house] the JO said, “: The circumstances that the decision-maker has reported [decision on house search] in this case cannot be considered sufficient grounds for reasonable suspicion that [X] had doping agents in the home at the time in question.”*

According to the respondent, this circumstance could have resulted in police officers not making this type of decision in future cases. Another respondent reported a barrier to testing urine samples, claiming that since individuals can refuse testing there is a need to implement mandatory testing, like with a catheter.

*Expand our coercive measures on those who are suspected, such as locking up or catheterizing those who refuse to urinate!*

Simplified tests were requested; for example, through blood tests or other rapid tests. Further, the relatively low penalty for doping offenses was mentioned as a barrier to the preventiontion efforts. Suggestions were made to change this rule to address the issue of doping. Moreover, participants found it difficult to calculate doping doses when reporting the use or handling of doping in connection with doping-related crimes because there are less-known substances found during house searches, which makes reporting of doping crimes time-consuming. According to the respondents, this, in combination with a relatively low penalty value compared with that for crimes related to narcotics and low knowledge of legislation around doping risks, can impede doping prevention activities.

*Penalty values are low. These factors, combined with the fact that far from all police officers have knowledge of, or routine use of doping legislation, risk creating a “vicious circle”. You do not report because you are unsure, you are unsure because you do not have sufficient knowledge or training*.

The Postal Act was also highlighted as a barrier to the detection of doping substances in postal items because the post staff are not allowed to open items owing to the low penalty value.

## 4. Discussion

This study examined police officers’ perceptions of doping, as it is defined in Swedish law; related problems; and suggestions for effective prevention of doping. Results showed that most respondents consider doping a societal problem and that doping occurs in combination with several other crimes, such as violence, illicit drug use, and drug dealing. These results are supported by a recent interview study with police officers in Sweden (34). Almost all respondents also believe it to be their duty to prevent doping but lack proper prerequisites to do so, emphasizing the opinion that there is a lack of knowledge in the police organization. This opinion was supported by the fact that approximately 75% of respondents reported that they lacked appropriate training to work against doping. More knowledge is, according to the respondents, perceived to be needed at all levels in the police organization; for example, municipalities police officers or intervention police officers must know how to recognize doping users and how to calculate doses when reporting suspected crimes. Further, investigation leaders, who give mandates for sampling and arrest, need to be properly informed about what is needed to facilitate the collection of evidence connected to doping offenses, according to the respondents. Additionally, respondents believe that managers in the police organization need to be better informed about doping to prioritize preventive activities. As previous implementation research supports the idea that sufficient knowledge is crucial for effective implementation (32, 33), the strengthening of basic education for police officers and further training within the police organization could abolish the barrier of ignorance (34).

Respondents further stated that quicker doping tests could accelerate the collection of evidence when building a doping offense case. Previous studies have with promising results assessed alternative test procedures, such as hair, saliva, or breath tests, which could make the collection of evidence more effective (36–38), however, to our knowledge, there are no commercially available instruments for testing via hair, saliva, or breath. Using a catheter to obtain urine samples from suspected individuals or using blood tests was also suggested by the respondents. However, this method is currently not allowed, at least not if coercion is needed, highlighting the relatively low penalty value for the doping use offense (28).

Good collaboration with gyms to facilitate police work was also emphasized by respondents as important for effective doping prevention. This coincides with a previous European study that emphasized collaboration between key actors to counteract doping among recreational athletes (39). A success factor in the current context, noted by respondents, was that local police districts appoint special liaison officers for gyms to communicate about doping-related issues. Collaboration agreements between gyms and the police, including enabling police officers to exercise undercover in the training premise to detect doping-related crimes, could also, according to the respondents, constitute facilitating factors. This view was previously highlighted in interviews with Swedish police officers (34). Several respondents also highlighted the importance of more gyms working actively against doping, preferably in line with the 100% PHT doping prevention method, which aligns with suggestions of field experts (10). This can include systematic policy work with clear routines for how gym staff handle different doping-related situations, staff communication with members that the gym takes a stand against doping, making information about the negative consequences of doping visible, and cooperation with the police (29).

Another barrier reported by respondents was the lack of economic and personnel resources to prevent doping. This barrier was previously highlighted in a study among key European actors in recreational sports (39), general implementation research (32), and a recent Swedish interview study among police officers (34). Thus, additional resources appear to be needed to increase the effectiveness of doping prevention. As previously indicated, increased knowledge about doping among managers in the police organization could amplify this priority (32, 33).

To decrease the availability of doping in society, several respondents highlighted the need to access postal items to detect doping substances. Currently, this is not permitted because doping offenses have relatively low penalty rates (28). Finally, suggestions for decreasing the doping demand in society; i.e., to convince people not to use doping substances, were put forward by respondents. These suggestions included engagement from other authorities and organizations, which coincides with previous research on the prevention of illicit substance use (10, 31, 39–42). Since doping has negative mental and physical health effects (11–20), healthcare sector professionals can play an important role in doping prevention in their encounters with users who could end up in psychiatry or other departments owing to doping side effects. Further, it is widely recognized that efforts to prevent harmful behaviors should be performed early in life. Therefore, schools and sports organizations with children and adolescents as pupils or members could be suitable areas for doping prevention interventions (43). Doping prevention in schools was recently supported by a systematic study of methods for preventing doping among young people (44); however, it has been difficult to indicate that the interventions changed students’ attitudes toward doping (45).

This study has several strengths that should be highlighted. It is based on responses from police officers from a wide range of regions in Sweden with various experience as police officers, different functions, and representing both men and women. An additional advantage is that the response rate is relatively high. The survey is also unique from a national and international perspective, as little has been published about police officers and their views on doping prevention.

This study also has certain limitations. The most prominent is the selection of respondents within the police authority or registered for a police training course on anti-doping work. This means that there could be a higher interest in addressing doping and related issues among respondents in this study than among police officers in general. However, only half the respondents reported knowing about the nationwide disseminated doping prevention method for 100% PHT, which indicates that several of them had low engagement in doping issues. Further, the structure of some of the response options was unbalanced with unequal numbers of positive and negative answer options, which could have contributed to response bias. Additionally, the instrument development, data collection, data interpretation, and data analysis were conducted by researchers who operate at a research and development unit whose practitioner group have been involved in the development of an anti-doping program. The respondents could also have fallen prey to social desirability bias (46). Although the proportion of women in our sample did not differ from the police force as a whole, the generalizability to the Swedish police force as a whole can be questioned as our sample was overrepresented in some regions and underrepresented in other regions. Moreover, the generalizability to the international context is partly limited since the Swedish context differs from most other countries regarding the prohibition of the use of doping substances. Finally, data collection took place during the COVID-19 pandemic, which affected the gyms’ operations and could have influenced respondents’ answers.

## 5. Conclusion

Police officers perceive doping to be a societal problem and are motivated to work against it. Those that participated in this study highlighted several areas for enhancing doping prevention, such as increasing knowledge in the police organization, legislative changes to enable simplified routines for doping tests, permission to control postal items, intensified doping prevention at gyms, and commitment from other authorities and organization to engage in doping prevention.

## Data Availability

The data are available from the Centre for Psychiatry Research, a collaboration between the Karolinska Institutet and Region Stockholm. However, restrictions apply to their availability, as they were used with ethical permission for this study and, therefore, are not publicly available. The data are available from the authors upon reasonable request and with permission from the Centre for Psychiatry Research.
